# Mathematical modeling and analysis of magnetic nanoparticle- induced heating in cerebrospinal fluid flow using a core–shell Fe_3_O_4_@Au nanoparticles for targeted drug therapy

**DOI:** 10.3389/fbioe.2026.1827203

**Published:** 2026-06-25

**Authors:** C. Helan Princey, A. David Maxim Gururaj

**Affiliations:** Department of Mathematics, School of Advanced Sciences, Vellore Institute of Technology, Chennai, Tamil Nadu, India

**Keywords:** cerebrospinal fluid flow, magnetic heating, magnetic nanoparticles, perturbation method, porous medium

## Abstract

**Background:**

Neurological disorders often require effective delivery of therapeutic agents to specific regions of the central nervous system. Magnetic nanoparticles have emerged as a promising approach for improving targeted drug delivery through cerebrospinal fluid (CSF) under externally applied magnetic fields. However, the combined effects of porous media, magnetic forces, nanoparticle transport, and magnetic heating on CSF flow remain insufficiently understood.

**Methods:**

In this study, a mathematical model is developed to investigate the flow and heat transfer characteristics of CSF containing Fe_3_O_4_@Au magnetic nanoparticles in a porous channel. The Brinkman--Darcy framework is employed to describe the flow, while magnetic body forces are incorporated through the Kelvin force model. Heat generation arising from the magnetic response of nanoparticles is included in the energy equation. The governing equations are transformed into dimensionless form and solved analytically using a perturbation technique to obtain expressions for velocity, temperature, volumetric flow rate, wall shear stress, and Nusselt number.

**Results:**

The analysis reveals that increasing permeability, Reynolds number, and magnetic interaction parameter enhances the velocity and volumetric flow rate of the nanofluid. In contrast, increasing nanoparticle volume fraction reduces fluid velocity due to the associated increase in effective viscosity. Temperature is found to increase significantly with magnetic heating, while higher thermal conductivity promotes heat diffusion and reduces thermal accumulation. The wall shear stress follows trends similar to velocity, increasing with permeability, Reynolds number, and magnetic forces. The Nusselt number is strongly influenced by magnetic heating and thermal conductivity, highlighting the competing effects of heat generation and heat diffusion.

**Discussion and Conclusion:**

The results demonstrate the significant role of magnetic forces, porous medium properties, and nanoparticle characteristics in controlling CSF transport and thermal behavior. The proposed model provides insight into the transport and distribution of magnetic nanoparticles in CSF and may contribute to the design and optimization of magnetically guided drug delivery systems for neurological applications.

## 1 Introduction

Cerebrospinal fluid (CSF) is essential in maintaining the health of the central nervous system, as it transports nutrients, eliminates waste, and protects neural tissues from damage. Intrathecal drug delivery, which means injecting drugs directly into the CSF has proven to be a successful treatment for neurological disorders. Neurological disorders represent a major global health challenge, affecting millions of people worldwide and leading to significant disability and mortality. According to the latest full analyses of the Global Burden of Disease (GBD) 2021 study, about 43% of the world’s population, or 3.4 billion people, had a condition affecting their nervous system (Steinmetz et al., 2024). This public health crisis kills 11.1 million people every year and is now the leading cause of health loss in the world, even more than cardiovascular diseases. There are a lot of different types of these disorders, such as neurodegenerative diseases like Alzheimer’s and Parkinson’s, cerebrovascular diseases like stroke, neurodevelopmental disorders like autism and neonatal encephalopathy, and infectious diseases like meningitis. Over 80% of neurological deaths happen in low- and middle-income countries, where medical care is limited and newborn conditions and infections are more common than they should be Solares et al. (2025).

Traditional treatment approaches for these conditions include pharmacological therapies, radiation therapy, and surgical interventions. Although these methods can help, they have important drawbacks. Surgical options (Feigin et al., 2019), like removing tumors or using deep brain stimulation (Benabid et al., 2009), are used when other treatments do not work. Neurorehabilitation helps improve thinking and movement by making use of the brain’s ability to adapt. Drug delivery to the brain is restricted by the blood–brain barrier, which prevents many therapeutic agents from reaching the target site effectively (Pardridge, 2012). Surgical procedures are invasive and carry risks, while radiotherapy may damage surrounding healthy tissues (Laperriere and Bernstein, 1994). As a result, achieving precise, targeted, and efficient treatment remains a major challenge.

To overcome these limitations, magnetic nanoparticles have emerged as a promising alternative in biomedical applications. Due to their small size and magnetic responsiveness, these nanoparticles can be guided externally to specific locations within the body, enabling targeted drug delivery and localized heating for therapeutic purposes (Pankhurst et al., 2003). This approach offers the potential to improve treatment efficiency while minimizing side effects, making it a valuable strategy for addressing neurological disorders. Core-shell nanoparticles, such as $Fe_{3}O_{4}@Au$ , provide an extremely versatile platform for this application (Laurent et al., 2008). The superparamagnetic $Fe_{3}O_{4}$ core enables precise spatiotemporal guidance via external magnetic fields and functions as a high-resolution contrast agent for Magnetic Resonance Imaging (MRI) (Gupta and Gupta, 2005). The gold (Au) shell provides essential biocompatibility, chemical stability, and a surface for functionalization with various ligands or antibodies, allowing for the direct delivery of therapeutic payloads to diseased brain regions (Dykman and Khlebtsov, 2017). Using the magnetoplasmonic properties of the CSF, $Fe_{3}O_{4}@Au$ nanoparticles can concentrate medications at specific lesion sites, overcoming the limitations of traditional systemic and local delivery while minimizing off-target toxicity (Kievit and Zhang, 2011). The United Nations’ Sustainable Development Goal 3 advocates for the utilization of safe and effective measures to enhance health and well-being. These modifications align with that objective. We need to understand how CSF flows and temperatures change when we use magnets to improve intrathecal drug delivery systems.

Magnetic nanoparticles are widely applied in biomedicine for magnetic separation, targeted drug and gene delivery, radio-frequency-induced hyperthermia, and magnetic resonance imaging, based on their controllable response to external magnetic fields (Pankhurst et al., 2003). Nanoparticle-based strategies offer promising solutions for central nervous system disorders by enabling drug transport across the blood–brain barrier through passive, receptor-mediated, and stimuli-responsive mechanisms (Toader et al., 2024). Numerical simulations showed that magnetite and maghemite nanoparticles can produce stable, controlled heating within tumors under safe magnetic field conditions, making them viable materials for magnetic hyperthermia Magnetic particle imaging is used in Banura et al. (2016) to estimate the specific absorption rate of magnetic nanoparticles and enable heat-transfer simulations for magnetic hyperthermia treatment planning. A finite-element magnetic fluid model was developed in Vizjak et al. (2021) to simulate magnetic hyperthermia by coupling electromagnetic steady-state and transient thermal calculations. The model links magnetic field parameters to specific absorption rate and temperature rise, enabling assessment and optimization of magnetic fluid heating performance. A numerical model was developed in Habibi and Ghasemi (2011) to study magnetically influenced transport of nanoparticles in a non-Newtonian biofluid by coupling Navier–Stokes, concentration, and magnetic field equations. The results showed that magnetic forces can overcome fluid drag, leading to strong nanoparticle accumulation near the magnetic source, which increases with magnetic field strength, particle size, and magnetic susceptibility. Finite element analysis has been widely applied to model magnetic nanoparticle hyperthermia, enabling simulation of nanoparticle behavior, heat generation, and heat transfer under alternating magnetic fields (Raouf et al., 2024).

A rapid, scalable flow-based synthesis method enables the formation of complete and uniform gold shells on magnetic nanoparticles, producing particles with combined magnetic and plasmonic properties (Mehdipour et al., 2022). ${\text{Fe}}_{3}{\text{O}}_{4}$ and ${\text{Fe}}_{3}{\text{O}}_{4}$ @C core–shell nanoparticle-based ferrofluids in Imran et al. (2021) exhibit significant enhancement in thermal conductivity compared to the base fluid, which increases with nanoparticle size and concentration. A finite-element mathematical model was developed in Gas et al. (2025) to simulate magnetic hyperthermia treatment of skin cancer using gold-coated iron oxide ( ${\text{Fe}}_{3}{\text{O}}_{4}$ @Au) magnetic nanoparticles based on coupled electromagnetic and bioheat equations. The simulations showed that heating efficiency and tumor damage strongly depend on blood perfusion and nanoparticle-induced heat generation, with maximum damage occurring at the tumor core. The increasing complexity of neurological disorders necessitates a departure from traditional treatment models constrained by the BBB and systemic toxicity (Pardridge, 2012). Conventional pharmacotherapy and surgery frequently fail to address underlying pathologies or pose significant risks (Kola and Landis, 2004). Magnetic nanoparticles, specifically core-shell $Fe_{3}O_{4}@Au$ formulations, provide a transformative alternative. They address the fundamental limitations of current therapies by allowing for precise magnetic targeting in the CSF and high-resolution theranostic capabilities (Mahmoudi et al., 2011; Xu et al., 2007). While challenges in neurotoxicity and clinical translation persist, the incorporation of MNPs into neurology heralds a new era of precision medicine capable of reducing the global burden of neurological disease.

The transport of cerebrospinal fluid (CSF) in the subarachnoid space can be effectively modeled using principles of fluid mechanics, where CSF is typically treated as a viscous fluid (Sweetman and Linninger, 2011) and, in advanced biomedical applications, as a nanofluid when therapeutic nanoparticles are introduced. Owing to the complex microstructure of the subarachnoid space, characterized by trabecular networks and irregular geometrical features, the flow domain is more appropriately represented as a porous medium (Helan Princey and David Maxim Gururaj, 2025; Linninger et al., 2024). In this context, Darcy’s law is commonly employed to describe the resistance offered by the porous structure, while its extension through the Brinkman model incorporates viscous shear effects (Nield and Bejan 2006). The resulting Brinkman–Darcy formulation provides a more realistic representation of CSF flow within such biological environments.

In the presence of magnetic nanoparticles, the application of a non-uniform external magnetic field gives rise to a magnetic body force, commonly referred to as the Kelvin force, which plays a crucial role in controlling nanoparticle transport. Unlike the classical Lorentz force, the Kelvin force arises due to magnetic field gradients and acts directly on the magnetization of the nanoparticles, enabling precise manipulation and targeting within the fluid domain (Davidson, 2017). This mechanism forms the basis for magnetic targeting in biomedical applications and significantly influences the momentum transport within the nanofluid (Pankhurst et al., 2003). In addition, thermal effects are of considerable importance, particularly in magnetic hyperthermia, where localized heating is induced for therapeutic purposes (Kumar and Mohammad, 2011). The inclusion of heat transfer, along with internal heat generation or absorption, allows for a comprehensive description of the energy dynamics in the system.

**FIGURE 1:**
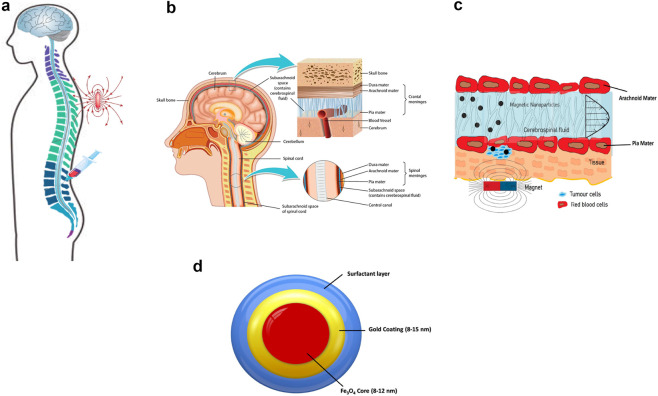
**(a)** Magnetic drug targeting. **(b)** Anatomy of the CSF-filled Subarachnoid Space. **(c)** Magnetic nanoparticles. **(d)** Structure of the Core Shell Nanoparticle ( ${\text{Fe}}_{3}{\text{O}}_{4}$ @Au).

To accurately characterize nanofluid behavior, effective thermophysical properties such as density, viscosity, thermal conductivity, and heat capacity are modeled as functions of the nanoparticle volume fraction, accounting for the combined influence of the base fluid and dispersed nanoparticles. Although numerous studies have investigated nanofluid flow, porous media transport, magnetic nanoparticle dynamics, and thermal effects in biomedical systems, these aspects are often examined in isolation. In particular, existing works on cerebrospinal fluid (CSF) flow, magnetic targeting, and nanoparticle-induced heat generation rarely consider their coupled interaction. There remains a significant lack of analytical models that simultaneously incorporate Brinkman–Darcy porous medium effects, Kelvin force-driven magnetic targeting, and heat generation under oscillating magnetic fields in CSF-based nanofluid systems. Consequently, the combined influence of these factors on flow behavior and temperature distribution is not yet fully understood. To address this gap, the present study develops a comprehensive coupled model integrating nanofluid dynamics, porous medium flow, magnetic forces, and thermal transport, providing deeper insights into targeted drug delivery (Figure 1a) and thermal therapy for neurological applications. The main objectives of this study are: 1. To develop a mathematical model for cerebrospinal fluid (CSF) flow containing magnetic nanoparticles in a porous channel. 2. To include the effect of magnetic forces acting on the nanoparticles and to incorporate heat generation due to nanoparticles using magnetic heating. 3. To convert the governing equations into non-dimensional form and solve the equations using the perturbation method. 4. To analyse the effects of physical parameters on velocity, temperature, flow rate, and heat transfer. 5. To understand how magnetic fields can influence nanoparticle transport and temperature in CSF.

## 2 Physical model and assumptions

This study examines the transport and heating of ${\text{Fe}}_{3}{\text{O}}_{4}$ @Au core-shell magnetic nanoparticles suspended in cerebrospinal fluid within the subarachnoid space. The subarachnoid space, bounded by the arachnoid and pia mater layers, carries a thin layer of cerebrospinal fluid (CSF) that circulates around the brain and spinal cord, as depicted in the Figure 1b. Cerebrospinal fluid (CSF) flow is modeled as flow through a porous channel because the region contains a complex network of trabeculae and collagen fibers (see Figure 1c). This approach makes it possible to include both viscous effects and resistance due to the porous structure (Darcy resistance) (Helan Princey and David Maxim Gururaj, 2025). The magnetic nanoparticles used in this study have a concentric core–shell structure, consisting of a magnetite ( ${\text{Fe}}_{3}{\text{O}}_{4}$ ) core coated with gold, as shown in Figure 1d.

### 2.1 Kelvin force modeling for magnetically guided nanoparticle transport

The motion of a magnetizable nanofluid in a porous medium is governed by the Brinkman–Darcy momentum equation augmented by a magnetic body force term. This model accounts for viscous diffusion within the porous structure (Brinkman term), flow resistance due to permeability (Darcy term), and magnetization effects arising from an externally applied non-uniform magnetic field.

The generalized vector form of the momentum equation for flow in a porous medium (Helan Princey and David Maxim Gururaj, 2025) with magnetic force is given by

$$\rho_{nf}\;\frac{\partial u^{*}}{\partial t^{*}}=-\nabla p^{*}+\mu_{nf}\;\nabla^{2}u^{*}-\frac{\mu_{nf}}{k}\;u^{*}+F_{\mathrm{mag}},$$ (1)

where $u^{*}$ is the velocity vector, $p^{*}$ is the pressure, $\rho_{nf}$ and $\mu_{nf}$ denote the effective density and viscosity of the nanofluid, k is the permeability of the porous medium, and $F_{\mathrm{mag}}$ represents the magnetic body force.

The magnetic force acting on a magnetizable nanofluid in a non-uniform magnetic field is best described by the Kelvin force, also called the magnetization force. This force occurs because the magnetic field strength changes from place to place, which causes the magnetic nanoparticles to become magnetized. In biomedical fluids containing particles such as ${\text{Fe}}_{3}{\text{O}}_{4}$ core–shell nanoparticles, the magnetic behavior depends on how the induced magnetic moments interact with the field gradient (Kayal et al., 2011).

Unlike electrically conducting fluids, where the Lorentz force is typically used, the applicability of Lorentz force in cerebrospinal fluid (CSF) requires careful consideration. Although CSF has a finite electrical conductivity (approximately 1.5–2.0 S/m), the contribution of induced currents depends on the magnetic Reynolds number, defined as $R_{m}=\mu_{0}\sigma UL$ , where σ is electrical conductivity, U is characteristic velocity, and L is characteristic length scale.

In typical CSF flow conditions, the characteristic velocity ( $U∼10^{-4}$ – $10^{-3}$ m/s) and length scale ( $L∼10^{-3}$ m) are very small, leading to $R_{m}\ll 1$ . For example, using σ≈2 S/m, the magnetic Reynolds number is of the order $10^{-9}$ – $10^{-7}$ . Such low values indicate that electromagnetic induction effects are negligible, and hence the induced current density remains very small. Consequently, the Lorentz force, which depends on induced currents, is insignificant compared to the magnetization force. This scaling argument is well established in magnetohydrodynamics and bioelectromagnetic systems (Davidson, 2017).

Furthermore, the heating mechanisms in magnetic nanoparticle systems are dominated by Néel and Brownian relaxation losses rather than eddy current heating, especially at the frequencies considered in biomedical applications (Rosensweig, 2002). Therefore, even though CSF is relatively more conductive than some biological tissues, the combined effects of low magnetic Reynolds number and nanoparticle-driven magnetization ensure that the Kelvin force remains the dominant magnetic body force. Hence, CSF with suspended magnetic nanoparticles behaves effectively as a weakly conducting but strongly magnetizable fluid. Under these conditions, the Kelvin force is more appropriate because it directly accounts for magnetization effects rather than induced electric currents. Therefore, the contribution of Lorentz force and eddy current effects is neglected in the present model.

Mathematically, the Kelvin force per unit volume is given by:

$$F_{\mathrm{mag}}=\mu_{0}\left(M\cdot \nabla \right)H$$

where $\mu_{0}$ is the magnetic permeability of free space, M is the magnetization, and H is the magnetic field strength. For linear magnetic materials, where magnetization is proportional to the magnetic field (M=χH) , this becomes:

$$F_{\mathrm{mag}}=\mu_{0}\chi \left(H\cdot \nabla \right)H$$

If the magnetic field varies only in one direction (for example, along the channel), the expression simplifies further to:

$$F_{\mathrm{mag}}=\mu_{0}\chi H\frac{\partial H}{\partial x}.$$

This result shows that motion of the particles and fluid occurs only when the magnetic field is non-uniform. This matches practical magnetic targeting methods, where external magnets create field gradients to guide nanoparticles toward specific locations such as tumors. The Kelvin force model is also suitable for low-speed flows and dilute nanoparticle suspensions, which are typical conditions in CSF flow. Therefore, including the Kelvin force in the momentum equation allows realistic modeling of magnetically guided drug delivery, localized hyperthermia, and controlled nanoparticle transport in the central nervous system.

To justify the assumption of negligible convective acceleration in the oscillatory flow regime, the Womersley number is evaluated. It is defined as

$$\alpha =d\sqrt{\frac{\omega \rho_{f}}{\mu_{f}}},$$

where d is the characteristic length, ω is the angular frequency, $\rho_{f}$ is the fluid density, and $\mu_{f}$ is the dynamic viscosity.

Using typical cerebrospinal fluid parameters ( $d∼10^{-3}$ m, $\rho_{f}∼10^{3}$ kg/m^3^, $\mu_{f}∼10^{-3}$ Pa ⋅ s, and ω∼2π rad/s), the Womersley number is estimated as α≈2 –3. This indicates a moderately unsteady but viscous-dominated flow. Furthermore, the Reynolds number remains very small (Re≪1) , confirming that inertial and convective effects are negligible. Therefore, the nonlinear convective term can be safely neglected in the present analysis.

It is also noted that the magnetic force formulation assumes a linear magnetic response of the nanoparticles, i.e., M=χH . In high magnetic field applications, magnetization may approach saturation and follow a nonlinear Langevin behavior.

To verify the validity of the linear approximation, the Langevin parameter is considered:

$$\xi =\frac{\mu_{p}H}{k_{B}T},$$

where $\mu_{p}$ is the magnetic moment of a nanoparticle, H is the magnetic field strength, $k_{B}$ is Boltzmann’s constant, and T is the absolute temperature.

Using typical values for ${\text{Fe}}_{3}{\text{O}}_{4}$ nanoparticles under biomedical conditions, the parameter is estimated to be ξ<1 , indicating that the system operates within the linear magnetization regime. Therefore, the approximation M=χH remains valid for the present study.

For significantly higher magnetic field strengths, nonlinear effects may become important, and the Langevin function would be required for more accurate modeling. Hence the above vector (Equation 1) reduces to the scalar dimensional form:

$$\rho_{nf}\;\frac{\partial u^{*}}{\partial t^{*}}=-\frac{\partial p^{*}}{\partial x^{*}}+\mu_{nf}\;\frac{\partial^{2}u^{*}}{\partial y^{*2}}-\frac{\mu_{nf}}{k}\;u^{*}+\mu_{0}\;\chi \;H^{*}\frac{\partial H^{*}}{\partial x^{*}}.$$ (2)

### 2.2 Energy equation with magnetic nanoparticle heating

The thermal behavior of the nanofluid is governed by the energy equation, which accounts for heat conduction within the fluid and internal heat generation due to magnetic nanoparticle heating. When magnetic nanoparticles are subjected to an alternating magnetic field, thermal energy is produced through magnetic relaxation mechanisms, and this effect is represented as a volumetric heat source term in the energy equation.

The dimensional energy equation for the nanofluid with magnetic heating can be written as

$${\left(\rho c_{p}\right)}_{nf}\;\frac{\partial T^{*}}{\partial t^{*}}=k_{nf}\;\nabla^{2}T^{*}+{\dot{q}\prime \prime \prime }_{\mathrm{mag}},$$ (3)

where ${\left(\rho c_{p}\right)}_{nf}$ is the effective heat capacity of the nanofluid, $k_{nf}$ is the effective thermal conductivity, $T^{*}$ is the temperature, and ${\dot{q}}_{\mathrm{mag}}^{\prime \prime \prime }$ represents the volumetric heat generation due to magnetic nanoparticles.

In many studies, heat generation is modeled using the Specific Absorption Rate (SAR), which is obtained experimentally. However, SAR is inherently dependent on the magnetic field frequency, nanoparticle properties, and relaxation mechanisms. Treating SAR as a constant does not fully capture these dependencies and limits the physical accuracy of the model.

To address this, the heat generation term is derived from electromagnetic principles and expressed in terms of the imaginary part of the complex magnetic susceptibility (Lodi et al., 2025). The volumetric heat generation due to magnetic nanoparticles is given by

$${\dot{q}}_{\mathrm{mag}}^{\prime \prime \prime }=\frac{1}{2}\mu_{0}\omega {H^{*}}^{2}\chi^{\prime \prime },$$ (4)

where $\mu_{0}$ is the magnetic permeability of free space, ω=2πf is the angular frequency of the applied magnetic field, $H^{*}$ is the magnetic field strength, and $\chi^{\prime \prime }$ is the imaginary part of the complex magnetic susceptibility.

The complex magnetic susceptibility is modeled using a Debye-type relaxation model, given by

$$\chi \left(\omega \right)=\frac{\chi_{0}}{1+i\omega \tau },$$

from which the imaginary component is obtained as

$$\chi^{\prime \prime }=\frac{\chi_{0}\;\omega \tau }{1+{\left(\omega \tau \right)}^{2}}.$$

here, $\chi_{0}$ is the static magnetic susceptibility and τ is the effective relaxation time, which accounts for both Néel and Brownian relaxation mechanisms. This formulation shows that heat generation depends explicitly on the frequency of the magnetic field and the relaxation behavior of the nanoparticles.

Substituting $\chi^{\prime \prime }$ into the heat generation term (Equation 4), we obtain:

$${\dot{q}}_{\mathrm{mag}}^{\prime \prime \prime }=\frac{1}{2}\mu_{0}\omega {H^{*}}^{2}\left(\frac{\chi_{0}\omega \tau }{1+{\left(\omega \tau \right)}^{2}}\right).$$

This expression demonstrates that the heat generation is frequency-dependent and reaches a maximum when ωτ≈1 , which corresponds to resonance between the applied magnetic field and nanoparticle relaxation.

The present formulation (Equation 3) provides a more physically consistent representation of magnetic heating compared to constant SAR models. It captures the influence of magnetic field strength, frequency, and nanoparticle properties, and is consistent with advanced electromagnetic models reported in the literature (Rosensweig, 2002).

Thus, replacing the SAR-based formulation with a susceptibility-based heat generation term improves the completeness and predictive capability of the model for biomedical applications such as magnetic drug targeting and controlled thermal therapy.

### 2.3 Assumption of uniform nanoparticle dispersion

In the present model, nanoparticles are assumed to be uniformly dispersed within the cerebrospinal fluid. This assumption simplifies the mathematical formulation and allows analytical treatment of the governing equations.

However, in realistic biomedical conditions, nanoparticle distribution is often non-uniform. Factors such as vascular transport, injection techniques, and interaction with biological tissues can lead to spatially heterogeneous distributions. Furthermore, nanoparticles may be sequestered by the immune system or accumulate preferentially in specific regions.

Another important consideration is nanoparticle aggregation. Magnetic nanoparticles tend to form clusters due to inter-particle magnetic dipole interactions. Such clustering can significantly influence both magnetic and thermal behavior. In particular, aggregation can lead to localized enhancement of heat generation, resulting in so-called thermal “hotspots,” which are critical in magnetic hyperthermia applications.

Moreover, clustering modifies the effective magnetic susceptibility of the nanoparticle system. Dipolar interactions between closely spaced particles alter the relaxation mechanisms (Neel and Brownian relaxation), which in turn affects the imaginary component of susceptibility and the resulting heat generation. As a consequence, the heating efficiency may either increase or decrease depending on the extent of aggregation.

Despite these complexities, the assumption of uniform dispersion is commonly adopted in theoretical models as a first approximation to capture the fundamental physics of magnetically induced transport and heating. The present model therefore provides a baseline understanding, while more advanced models incorporating spatial heterogeneity and particle interactions can be considered in future work.

#### 2.3.1 Nanofluid thermophysical properties

The effective thermophysical properties of the nanofluid are modeled using classical mixture relations. The density of the nanofluid is expressed as:

$$\rho_{nf}=\left(1-\phi \right)\rho_{f}+\phi \rho_{np}$$

The effective dynamic viscosity is given by Brinkman’s model:

$$\mu_{nf}=\mu_{f}{\left(1-\phi \right)}^{-2.5}$$

The heat capacity of the nanofluid is defined as:

$${\left(\rho C_{p}\right)}_{nf}=\left(1-\phi \right){\left(\rho C_{p}\right)}_{f}+\phi {\left(\rho C_{p}\right)}_{np}$$

The effective thermal conductivity is evaluated using the Maxwell model:

$$k_{nf}=k_{f}\left[\frac{k_{np}+2k_{f}-2\phi \left(k_{f}-k_{np}\right)}{k_{np}+2k_{f}+\phi \left(k_{f}-k_{np}\right)}\right]$$

## 3 Mathematical formulation

We consider unsteady flow and heat transfer of a nanofluid representing cerebrospinal fluid (CSF) containing ${\text{Fe}}_{3}{\text{O}}_{4}$ @Au magnetic core–shell nanoparticles in a two-dimensional porous channel. The flow is driven by a pressure gradient and influenced by an externally applied non-uniform magnetic field. Heat generation within the nanofluid arises from the magnetic response of the nanoparticles under an alternating magnetic field and is modeled through the frequency-dependent imaginary component of the magnetic susceptibility.

The following assumptions are adopted to simplify the physical problem:

- The flow is laminar, incompressible, and Newtonian fluid flowing along the axial direction (Thomas, 2019).
- The nanofluid behaves as a single-phase homogeneous fluid with effective thermophysical properties.
- Nanoparticles are uniformly dispersed and remain in thermal equilibrium with the base fluid.
- The porous medium representing the subarachnoid space obeys the Brinkman–Darcy model.
- Heat generation results from magnetic relaxation losses and is modeled using the imaginary component of the complex magnetic susceptibility, thereby accounting for the frequency‐dependent heating behavior of the nanoparticles.
- Fluid properties are constant except where modified by nanoparticle concentration.

A Cartesian coordinate system is chosen such that the x -axis lies along the channel length and the y -axis is normal to the walls.

### 3.1 Governing equations

The flow of a magnetized nanofluid in a porous medium is governed by the modified incompressible Navier–Stokes equation incorporating Darcy resistance and magnetic body force effects (Equation 2). Under the assumption of low Reynolds number flow, the nonlinear convective terms are neglected. The dimensional momentum equation is given by:

$$\left[\left(1-\phi \right)\rho_{f}+\phi \rho_{np}\right]\frac{\partial u^{*}}{\partial t^{*}}=-\frac{\partial p^{*}}{\partial x^{*}}+\mu_{f}{\left(1-\phi \right)}^{-2.5}\frac{\partial^{2}u^{*}}{\partial y^{*2}}-\frac{\mu_{f}{\left(1-\phi \right)}^{-2.5}}{k}u^{*}+\mu_{0}\chi H^{*}\frac{\partial H^{*}}{\partial x^{*}}$$ (5)

The energy transport within the nanofluid, accounting for thermal conduction and magnetic hyperthermia heating, is described by:

$$\left[\left(1-\phi \right){\left(\rho C_{p}\right)}_{f}+\phi {\left(\rho C_{p}\right)}_{np}\right]\frac{\partial T^{*}}{\partial t^{*}}=k_{f}\left[\frac{k_{np}+2k_{f}-2\phi \left(k_{f}-k_{np}\right)}{k_{np}+2k_{f}+\phi \left(k_{f}-k_{np}\right)}\right]\frac{\partial^{2}T^{*}}{\partial y^{*2}}+\frac{1}{2}\mu_{0}\omega H^{*2}\left(\frac{\chi_{0}\omega \tau }{1+{\left(\omega \tau \right)}^{2}}\right)$$ (6)

The flow is considered between two parallel plates separated by a distance d . The no-slip condition is imposed on the velocity at both walls, while the temperature is prescribed at the boundaries.

The fluid satisfies the no-slip condition at both channel walls:

$$u^{*}\left(0,t^{*}\right)=0,\;u^{*}\left(d,t^{*}\right)=0$$

The lower wall is maintained at a reference temperature $T_{0}$ , while the upper wall is maintained at a higher temperature $T_{w}$ :

$$T^{*}\left(0,t^{*}\right)=T_{0},\;T^{*}\left(d,t^{*}\right)=T_{w}$$

The flow is driven by an oscillatory pressure gradient applied along the axial direction, given by:

$$\frac{\partial p^{*}}{\partial x^{*}}=-\lambda e^{i\omega t^{*}}$$

A non-uniform magnetic field is imposed along the flow direction, represented as:

$$H^{*}=H^{*}\left(x^{*}\right)$$

### 3.2 Non-dimensionalisation

To reduce the governing equations to a dimensionless form and identify the dominant physical parameters, the following characteristic scales are introduced. The spatial coordinates are scaled with the characteristic length d , while the velocity and time are scaled using the characteristic velocity $U_{0}$ :

$$x=\frac{x^{*}}{d},\;y=\frac{y^{*}}{d},\;u=\frac{u^{*}}{U_{0}},\;t=\frac{t^{*}U_{0}}{d}$$

The temperature, magnetic field, and pressure are non-dimensionalised as:

$$\theta =\frac{T^{*}-T_{0}}{\Delta T},\;H=\frac{H^{*}}{H_{0}},\;p=\frac{p^{*}}{\rho_{f}U_{0}^{2}}$$

The following dimensionless parameters arise naturally from the scaling process. The Reynolds number (Re) represents the ratio of inertial to viscous forces, while the Peclet number (Pe) characterizes the relative importance of convective to conductive heat transfer:

$$Re=\frac{\rho_{f}U_{0}d}{\mu_{f}},\;Pe=\frac{\rho_{f}C_{p,f}U_{0}d}{k_{f}}$$

To incorporate the effects of nanoparticle volume fraction, the effective thermophysical properties of the nanofluid are expressed in dimensionless form as ratios relative to the base fluid:

$$\Phi_{\rho }=\frac{\rho_{nf}}{\rho_{f}},\;\Phi_{\mu }=\frac{\mu_{nf}}{\mu_{f}},\;\Phi_{\left(\rho C_{p}\right)}=\frac{{\left(\rho C_{p}\right)}_{nf}}{{\left(\rho C_{p}\right)}_{f}}$$

The permeability of the porous medium is represented through the Darcy number:

$$K=\frac{k}{d^{2}}$$

The ratio of effective thermal conductivity of the nanofluid to that of the base fluid is defined as:

$$\lambda_{n}=\frac{k_{nf}}{k_{f}}$$

The magnetic interaction parameter, which quantifies the influence of the applied magnetic field on the flow, is given by:

$$S=\frac{\mu_{0}\chi H_{0}^{2}}{\rho_{f}U_{0}^{2}}$$

The dimensionless heat generation parameter due to magnetic nanoparticle heating is defined as:

$$Q^{*}=\frac{\mu_{0}\omega H_{0}^{2}d^{2}}{2k_{f}\Delta T}\cdot \frac{\chi_{0}\omega \tau }{1+{\left(\omega \tau \right)}^{2}}$$

### 3.3 Dimensionless governing equations

By substituting the above non-dimensional variables into the dimensional governing (Equations 5,6) and simplifying, the following dimensionless forms are obtained. The dimensionless momentum equation is expressed as

$$\Phi_{\rho }\frac{\partial u}{\partial t}=-\frac{\partial p}{\partial x}+\frac{\Phi_{\mu }}{Re}\frac{\partial^{2}u}{\partial y^{2}}-\frac{\Phi_{\mu }}{K\;Re}u+S\;H\frac{\partial H}{\partial x}$$ (7)

The corresponding dimensionless energy equation, incorporating magnetic hyperthermia heating, is given by:

$$\Phi_{\left(\rho C_{p}\right)}Pe\frac{\partial \theta }{\partial t}=\lambda_{n}\frac{\partial^{2}\theta }{\partial y^{2}}+Q^{*}H^{2}$$ (8)

Using the dimensionless variables defined earlier, the governing (Equations 7, 8) are solved subject to the following boundary conditions. The no-slip condition at the channel walls yields:

$$u\left(0,t\right)=0,\;u\left(1,t\right)=0$$

The lower and upper walls are maintained at constant temperatures, which in dimensionless form become:

$$\theta \left(0,t\right)=0,\;\theta \left(1,t\right)=1$$

The dimensionless form of the oscillatory pressure gradient is given by:

$$\frac{\partial p}{\partial x}=-\lambda e^{i\omega t}$$

## 4 Perturbation solution

To obtain analytical solutions for the velocity and temperature fields, a perturbation approach is employed under the assumption of small oscillatory forcing. The flow is driven by an oscillatory pressure gradient, which introduces time-dependent behavior into the system.

The velocity and temperature fields are expanded using a regular perturbation method as follows:

$$\begin{array}{c} u\left(y,t\right)=u_{0}\left(y\right)+\varepsilon u_{1}\left(y\right)e^{i\omega t}\\ \theta \left(y,t\right)=\theta_{0}\left(y\right)+\varepsilon \theta_{1}\left(y\right)e^{i\omega t} \end{array}$$ (9)

where ε≪1 is a small perturbation parameter. The functions $u_{0}\left(y\right)$ and $\theta_{0}\left(y\right)$ represent the steady components, while $u_{1}\left(y\right)$ and $\theta_{1}\left(y\right)$ correspond to the oscillatory components.

Upon applying the perturbation expansion, the boundary conditions for the zeroth- and first-order components are obtained as follows:

$$\begin{array}{c} u_{0}\left(0\right)=0,\;u_{0}\left(1\right)=0,\;u_{1}\left(0\right)=0,\;u_{1}\left(1\right)=0\\ \theta_{0}\left(0\right)=0,\;\theta_{0}\left(1\right)=1,\;\theta_{1}\left(0\right)=0,\;\theta_{1}\left(1\right)=1 \end{array}$$ (10)

Substituting the perturbation expansions (Equations 9, 10) into the governing equations and collecting terms of order O(1) yields the steady-state equations.

$$\frac{d^{2}u_{0}}{dy^{2}}-\frac{1}{K}u_{0}=-\frac{Re}{\Phi_{\mu }}SH\frac{dH}{dx}$$

Applying the boundary conditions, the steady component of velocity is given by:

$$u_{0}\left(y\right)=A\left[1+\frac{\left(e^{-\alpha }-1\right)e^{\alpha y}+\left(1-e^{\alpha }\right)e^{-\alpha y}}{e^{\alpha }-e^{-\alpha }}\right]$$ (11)

where

$$\alpha =\frac{1}{\sqrt{K}},\;A=K\frac{Re}{\Phi_{\mu }}SH\frac{dH}{dx}$$

Collecting terms of order O(ε) yields the oscillatory components.

$$\frac{d^{2}u_{1}}{dy^{2}}-\left(\frac{1}{K}+\frac{Re\Phi_{\rho }i\omega }{\Phi_{\mu }}\right)u_{1}=-\frac{Re}{\Phi_{\mu }}\lambda$$

Let

$$M^{2}=\frac{1}{K}+\frac{Re\Phi_{\rho }i\omega }{\Phi_{\mu }}$$

Applying boundary conditions, the oscillatory component of velocity is:

$$u_{1}\left(y\right)=B\left[1+\frac{\left(e^{-M}-1\right)e^{My}+\left(1-e^{M}\right)e^{-My}}{e^{M}-e^{-M}}\right]$$ (12)

where

$$M^{2}=\frac{1}{K}+\frac{Re\Phi_{\rho }i\omega }{\Phi_{\mu }},\;B=\frac{Re}{\Phi_{\mu }}\frac{\lambda }{M^{2}}$$

Now, for the temperature field,

$$\lambda_{n}\frac{d^{2}\theta_{0}}{dy^{2}}+Q^{*}H^{2}=0$$

Integrating twice and applying the boundary conditions $\theta_{0}\left(0\right)=0$ and $\theta_{0}\left(1\right)=1$ , the steady temperature distribution is obtained as:

$$\theta_{0}\left(y\right)=-\frac{Q^{*}H^{2}}{2\lambda_{n}}y^{2}+\left(1+\frac{Q^{*}H^{2}}{2\lambda_{n}}\right)y$$ (13)

Collecting terms of order O(ε) yields the oscillatory components.

$$\lambda_{n}\frac{d^{2}\theta_{1}}{dy^{2}}-i\omega \Phi_{\left(\rho C_{p}\right)}Pe\;\theta_{1}=0$$

Defining:

$$N^{2}=\frac{i\omega \Phi_{\left(\rho C_{p}\right)}Pe}{\lambda_{n}}$$

Applying the boundary conditions $\theta_{1}\left(0\right)=0$ and $\theta_{1}\left(1\right)=1$ , we obtain:

$$\theta_{1}\left(y\right)=A\left[1-\cosh\left(Ny\right)+\frac{\cosh\left(N\right)-1}{\sinh\left(N\right)}\sinh\left(Ny\right)\right]$$ (14)

The complete velocity and temperature fields (using Equations 11–14) are given by:

$$u\left(y,t\right)=A\left[1+\frac{\left(e^{-\alpha }-1\right)e^{\alpha y}+\left(1-e^{\alpha }\right)e^{-\alpha y}}{e^{\alpha }-e^{-\alpha }}\right]+\varepsilon e^{i\omega t}\;B\left[1+\frac{\left(e^{-M}-1\right)e^{My}+\left(1-e^{M}\right)e^{-My}}{e^{M}-e^{-M}}\right]$$ (15)

where

$$A=K\frac{Re}{\Phi_{\mu }}SHH_{x},\;B=\frac{Re}{\Phi_{\mu }}\frac{\lambda }{M^{2}},\;\alpha =\frac{1}{\sqrt{K}},\;M^{2}=\frac{1}{K}+\frac{Re\Phi_{\rho }i\omega }{\Phi_{\mu }}.$$

$$\theta \left(y,t\right)=y+\frac{Q^{*}H^{2}}{2\lambda_{n}}\left(y-y^{2}\right)+\varepsilon e^{i\Omega t}A\left[1-\cosh\left(Ny\right)+\frac{\cosh N-1}{\sinh N}\sinh\left(Ny\right)\right]$$ (16)

where

$$Q^{*}=\frac{\mu_{0}\omega H_{0}^{2}d^{2}}{2k_{f}\Delta T}\cdot \frac{\chi_{0}\omega \tau }{1+{\left(\omega \tau \right)}^{2}},\;N^{2}=\frac{i\omega \Phi_{\left(\rho C_{p}\right)}Pe}{\lambda_{n}}.$$

### 4.1 Nusselt number

The dimensionless temperature field is given by:

$$\theta \left(y,t\right)=y+\frac{Q^{*}H^{2}}{2\lambda_{n}}\left(y-y^{2}\right)+\varepsilon e^{i\Omega t}A\left[1-\cosh\left(Ny\right)+\frac{\cosh N-1}{\sinh N}\sinh\left(Ny\right)\right]$$

The Nusselt number is defined as:

$$Nu=-\frac{\partial \theta }{\partial y}$$ (17)

Differentiating the temperature field with respect to y :

$$\frac{\partial \theta }{\partial y}=1+\frac{Q^{*}H^{2}}{2\lambda_{n}}\left(1-2y\right)+\varepsilon e^{i\Omega t}A\left[-N\sinh\left(Ny\right)+\frac{\cosh N-1}{\sinh N}N\cosh\left(Ny\right)\right]$$

Substituting the above equation in Equation 17:

$$Nu\left(y,t\right)=-[1+\frac{Q^{*}H^{2}}{2\lambda_{n}}\left(1-2y\right)+\varepsilon e^{i\Omega t}A\left(-N\sinh\left(Ny\right)+\frac{\cosh N-1}{\sinh N}N\cosh\left(Ny\right)\right)]$$ (18)

The Nusselt Number at the Lower Wall (y=0) is given by

$$Nu_{0}=-\left[1+\frac{Q^{*}H^{2}}{2\lambda_{n}}+\varepsilon e^{i\Omega t}A\frac{\left(\cosh N-1\right)}{\sinh N}N\right]$$

The Nusselt Number at the Upper Wall (y=1) is given by

$$Nu_{1}=-\left[1-\frac{Q^{*}H^{2}}{2\lambda_{n}}+\varepsilon e^{i\Omega t}A\left(-N\sinh N+\frac{\cosh N-1}{\sinh N}N\cosh N\right)\right]$$

### 4.2 Flow rate

The volumetric flow rate per unit width, denoted by Q(t) , is defined as the integral of the velocity profile across the channel height:

$$Q\left(t\right)=\int_{0}^{1}u\left(y,t\right)\;dy$$ (19)

Substituting the obtained velocity field:

$$u\left(y,t\right)=u_{0}\left(y\right)+\varepsilon u_{1}\left(y\right)e^{i\omega t}$$

the flow rate (Equation 19) can be expressed as:

$$Q\left(t\right)=Q_{0}+\varepsilon e^{i\omega t}Q_{1}$$

where

$$Q_{0}=\int_{0}^{1}u_{0}\left(y\right)\;dy,Q_{1}=\int_{0}^{1}u_{1}\left(y\right)\;dy$$

$$Q_{0}=A\left[1+\frac{\left(e^{-\alpha }-1\right)\left(e^{\alpha }-1\right)}{\alpha \left(e^{\alpha }-e^{-\alpha }\right)}+\frac{\left(1-e^{\alpha }\right)\left(1-e^{-\alpha }\right)}{\alpha \left(e^{\alpha }-e^{-\alpha }\right)}\right]$$

$$Q_{1}=B\left[1+\frac{\left(e^{-M}-1\right)\left(e^{M}-1\right)}{M\left(e^{M}-e^{-M}\right)}+\frac{\left(1-e^{M}\right)\left(1-e^{-M}\right)}{M\left(e^{M}-e^{-M}\right)}\right]$$

Substituting $Q_{0}$ and $Q_{1}$ , the complete expression for flow rate is:

$$Q\left(t\right)=A\left[1+\frac{4-2\left(e^{\alpha }+e^{-\alpha }\right)}{\alpha \left(e^{\alpha }-e^{-\alpha }\right)}\right]+\varepsilon e^{i\omega t}\;B\left[1+\frac{4-2\left(e^{M}+e^{-M}\right)}{M\left(e^{M}-e^{-M}\right)}\right]$$ (20)

### 4.3 Wall shear stress

The wall shear stress is defined as the gradient of velocity at the wall. In dimensionless form, it is given by:

$$\tau_{w}={\left.\frac{\partial u}{\partial y}\right|}_{\text{wall}}$$ (21)

The wall shear stress (Equation 21) at y=0 is given by:

$$\tau_{w}=K\frac{Re}{\Phi_{\mu }}SHH_{x}\;\alpha \frac{e^{\alpha }+e^{-\alpha }-2}{e^{\alpha }-e^{-\alpha }}+\varepsilon e^{i\omega t}\left[\frac{Re}{\Phi_{\mu }}\frac{\lambda }{M^{2}}\right]M\frac{e^{M}+e^{-M}-2}{e^{M}-e^{-M}}$$

$$\tau_{w}=K\frac{Re}{\Phi_{\mu }}SHH_{x}\;\alpha \frac{e^{\alpha }+e^{-\alpha }-2}{e^{\alpha }-e^{-\alpha }}+\varepsilon e^{i\omega t}\frac{Re\lambda }{\Phi_{\mu }M}\frac{e^{M}+e^{-M}-2}{e^{M}-e^{-M}}$$ (22)

where

$$\alpha =\frac{1}{\sqrt{K}},\;M^{2}=\frac{1}{K}+\frac{Re\Phi_{\rho }i\omega }{\Phi_{\mu }}$$

## 5 Results and discussion

In this section, the effects of key governing parameters on the velocity, temperature, Nusselt number, flow rate, and wall shear stress are analyzed. The analytical solutions obtained in the previous sections are used to investigate the influence of nanoparticle volume fraction, magnetic field strength, Peclet number, and oscillation frequency.

### 5.1 Velocity profile

The velocity field (Equation 15) consists of both steady and oscillatory components. The steady component is primarily governed by the magnetic interaction parameter and permeability, while the oscillatory component is influenced by the pressure gradient amplitude and frequency through the complex parameter M .

**FIGURE 2:**
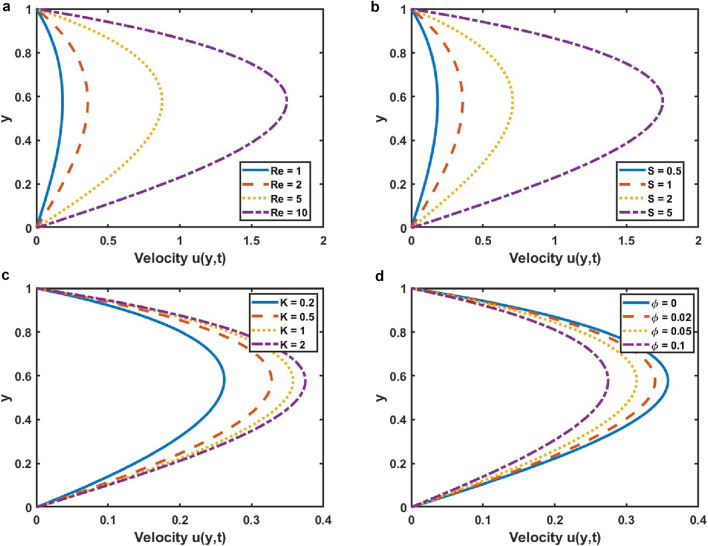
**(a)** Variation of velocity for different values of Re . **(b)** Variation of velocity for different values of S . **(c)** Variation of velocity for different values of K . **(d)** Variation of velocity for different values of ϕ .

It is observed that the velocity increases with increasing Reynolds number (see Figure 2a). Higher values of Re indicate stronger inertial effects compared to viscous forces, which enhances the fluid motion. As a result, the peak velocity inside the channel becomes larger, while the no-slip condition at the walls remains satisfied. This behaviour is consistent with classical fluid dynamics, where an increase in Reynolds number leads to a higher flow rate.

The influence of the permeability parameter K on the velocity profile shows that velocity increases significantly as K increases (see Figure 2c). Physically, higher permeability reduces the resistance offered by the porous medium, allowing the fluid to pass more easily through the channel. For smaller values of K, the porous medium strongly restricts the flow, leading to lower velocities throughout the domain. This confirms the dominant role of Darcy resistance in controlling the flow behaviour.

The velocity profile increases with increasing magnetic interaction parameter S (see Figure 2b). When S = 0, the flow is purely pressure-driven. As S increases, the Kelvin force generated by the non-uniform magnetic field acts as an additional driving force, accelerating the fluid. This leads to a noticeable increase in velocity across the channel. The results clearly demonstrate that magnetic fields can be effectively used to control and enhance fluid motion in nanoparticle-based systems.

The velocity decreases with increasing nanoparticle volume fraction ϕ (see Figure 2d). This is mainly due to the increase in effective viscosity of the nanofluid, which introduces greater resistance to flow. Although the presence of nanoparticles enhances thermal properties, it negatively affects the momentum transport by slowing down the fluid. This indicates a trade-off between thermal enhancement and flow efficiency.

From the above results, it is clear that the velocity of the nanofluid is influenced by a combination of hydrodynamic, porous, and magnetic effects. The Reynolds number and permeability parameter enhance the flow, while the magnetic interaction parameter provides an additional controllable driving force. In contrast, increasing nanoparticle concentration reduces the velocity due to increased viscosity. These results are important for biomedical applications, as they show that fluid transport can be controlled using magnetic fields, while also highlighting the need to balance nanoparticle concentration to avoid excessive flow resistance.

### 5.2 Temperature distribution

The temperature field (Equation 16) consists of a steady parabolic component and an oscillatory component. The steady temperature distribution is governed by the magnetic heat generation parameter $Q^{*}$ and the effective thermal conductivity ratio $\lambda_{n}$ .

**FIGURE 3:**
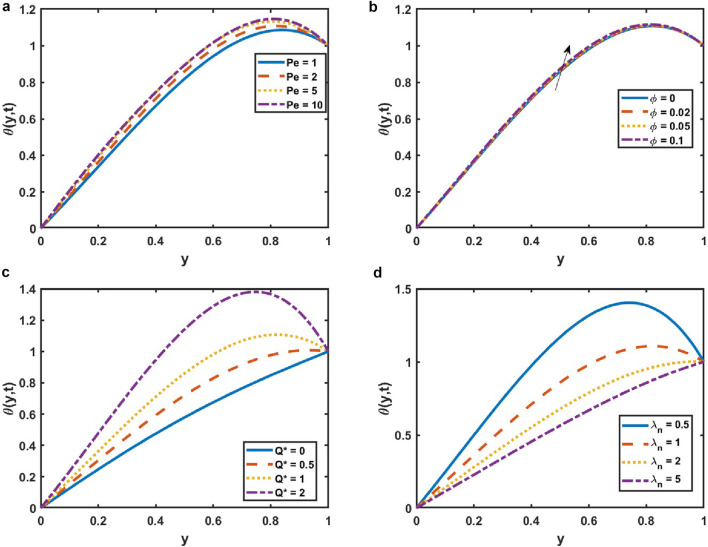
**(a)** Variation of temperature for different values of Pe . **(b)** Variation of temperature for different values of ϕ . **(c)** Variation of temperature for different values of Q . **(d)** Variation of temperature for different values of $\lambda_{n}$ .

Figure 3a illustrates the influence of the Peclet number Pe on temperature distribution. It is observed that the effect of Pe on temperature is relatively weak in the present model. A slight increase in temperature is seen with increasing Pe, which indicates a marginal dominance of convective transport over diffusion. However, since the energy equation is dominated by conduction and internal heat generation, the influence of Pe remains limited.

Figure 3b shows the effect of nanoparticle volume fraction ϕ on temperature distribution. It is observed that the temperature variation with ϕ is relatively small. A slight increase in temperature is seen as ϕ increases. This behavior is due to competing effects. Increasing ϕ enhances thermal conductivity, which promotes heat diffusion, while also increasing the heat capacity of the nanofluid. These effects partially balance each other, resulting in only a mild change in temperature.

The influence of the magnetic heating parameter $Q^{*}$ on the temperature distribution is shown in Figure 3c. It is observed that the temperature increases significantly with increasing $Q^{*}$ . This is because $Q^{*}$ represents internal heat generation due to magnetic nanoparticles. As $Q^{*}$ increases, more thermal energy is generated within the fluid, resulting in higher temperatures throughout the channel. The temperature profile becomes more curved (parabolic), indicating stronger internal heating effects. The maximum temperature occurs within the domain rather than at the boundaries, confirming volumetric heat generation.

The effect of the thermal conductivity ratio $\lambda_{n}$ is presented in Figure 3d. It is clearly observed that the temperature decreases as $\lambda_{n}$ increases. Higher thermal conductivity enhances heat diffusion, allowing heat to spread more efficiently throughout the fluid. As a result, the temperature profile becomes flatter and the peak temperature decreases. This indicates that stronger conductive effects reduce thermal accumulation within the domain.

From the above results, it is evident that temperature distribution is primarily governed by internal heat generation and thermal conductivity. The magnetic heating parameter $Q^{*}$ plays a dominant role in increasing temperature, while thermal conductivity acts to diffuse heat and reduce temperature gradients. The nanoparticle volume fraction and Peclet number have comparatively weaker effects. Overall, the temperature profiles confirm that the system is strongly influenced by magnetic heating and conductive transport mechanisms.

### 5.3 Nusselt number

**FIGURE 4:**
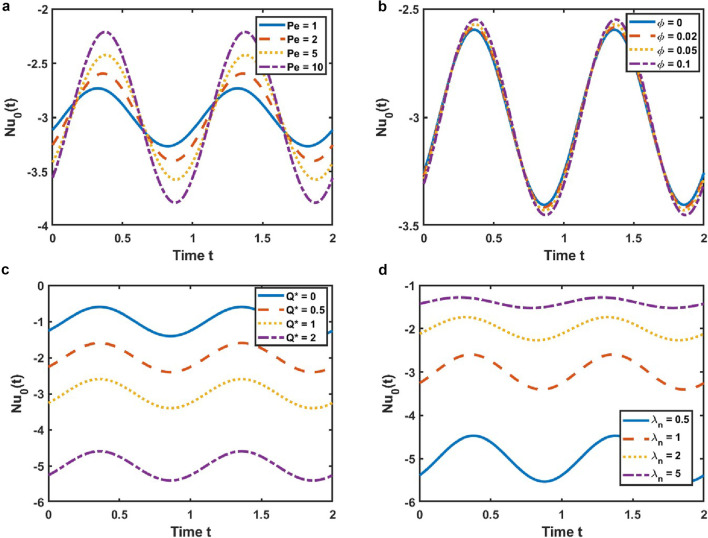
**(a)** Variation of Nusselt Number for different values of Pe . **(b)** Variation of Nusselt Number for different values of Φ . **(c)** Variation of Nusselt Number for different values of Q* . **(d)** Variation of Nusselt Number for different values of λ *_n_*.

The Nusselt number (Equation 18) represents the rate of heat transfer at the channel walls. The derived expressions show that it consists of both steady and oscillatory contributions. The variation of the Nusselt number with Peclet number Pe is presented in Figure 4a. It is observed that increasing Pe leads to a noticeable increase in the amplitude of oscillations in the Nusselt number. This indicates that convective effects become more significant at higher Pe, enhancing the time-dependent variation of heat transfer. While the average value does not change drastically, the fluctuation in heat transfer becomes more pronounced.

Figure 4b illustrates the effect of nanoparticle volume fraction ϕ on the Nusselt number. It is observed that the influence of ϕ is relatively weak compared to other parameters. A slight variation in the Nusselt number is observed as ϕ increases. This is due to competing effects: the addition of nanoparticles increases thermal conductivity, which tends to enhance heat transfer, while also increasing heat capacity, which tends to reduce temperature gradients. These competing mechanisms result in only minor changes in the Nusselt number.

The variation of the Nusselt number Nu(t) with time for different values of the magnetic heating parameter $Q^{*}$ is shown in Figure 4c. It is observed that the Nusselt number decreases (becomes more negative) as $Q^{*}$ increases. This behavior indicates that increasing internal heat generation reduces the temperature gradient at the wall. Since the Nusselt number represents the rate of heat transfer at the wall, a reduction in its magnitude implies weaker heat removal due to the accumulation of heat within the fluid. Although the oscillatory nature remains unchanged, the entire profile shifts downward with increasing $Q^{*}$ , confirming that magnetic heating reduces wall heat transfer.

Figure 4d shows the effect of thermal conductivity ratio $\lambda_{n}$ on the Nusselt number. It is clearly observed that the Nusselt number increases with increasing $\lambda_{n}$ . Higher thermal conductivity enhances heat diffusion toward the wall, resulting in a steeper temperature gradient and thus higher heat transfer rates. As a result, the Nusselt number increases significantly with $\lambda_{n}$ .

From the above results, it is evident that the Nusselt number is primarily influenced by internal heat generation and thermal conductivity. The magnetic heating parameter $Q^{*}$ reduces the wall heat transfer by increasing internal thermal energy, while higher thermal conductivity enhances heat transfer. The effects of nanoparticle volume fraction and Peclet number are comparatively weaker, although Peclet number influences the oscillatory behavior of heat transfer. Overall, the results highlight the competing roles of heat generation and heat diffusion in determining thermal transport characteristics.

The sensitivity of the Nusselt number to uncertainties in nanoparticle geometry is examined by varying the Au-shell thickness of ${\text{Fe}}_{3}{\text{O}}_{4}$ @Au nanoparticles. The shell thickness directly influences the effective thermal conductivity of the nanoparticles, which in turn modifies the nanofluid conductivity and the dimensionless parameter $\lambda_{n}$ .

As shown in Table 1, increasing the shell thickness leads to a significant increase in effective thermal conductivity due to the high conductivity of gold. This results in higher values of $\lambda_{n}$ , which reduces the magnitude of the term $\frac{Q^{*}}{2\lambda_{n}}$ in the Nusselt number expression.

**TABLE 1:** Sensitivity of Nusselt number to Au-shell thickness through thermal conductivity.

Shell thickness (nm)	$k_{np,eff}$ (W/mK)	$\lambda_{n}=\frac{k_{nf}}{k_{f}}$	$Nu_{0}$
0	6	1.0	−2.00
2	20	1.5	−1.67
5	60	2.5	−1.40
10	150	4.0	−1.25

Consequently, the Nusselt number increases (becomes less negative), indicating improved heat transfer at the wall. This demonstrates that uncertainties in Au-shell thickness can directly affect thermal predictions, highlighting the importance of accurate nanoparticle characterization in magnetic heating applications.

### 5.4 Flow rate

The volumetric flow rate (Equation 20) comprises steady and oscillatory components. The steady flow rate increases with the magnetic parameter S , as the magnetic body force enhances fluid motion. However, increased resistance due to the porous medium reduces the overall flow rate.

**FIGURE 5:**
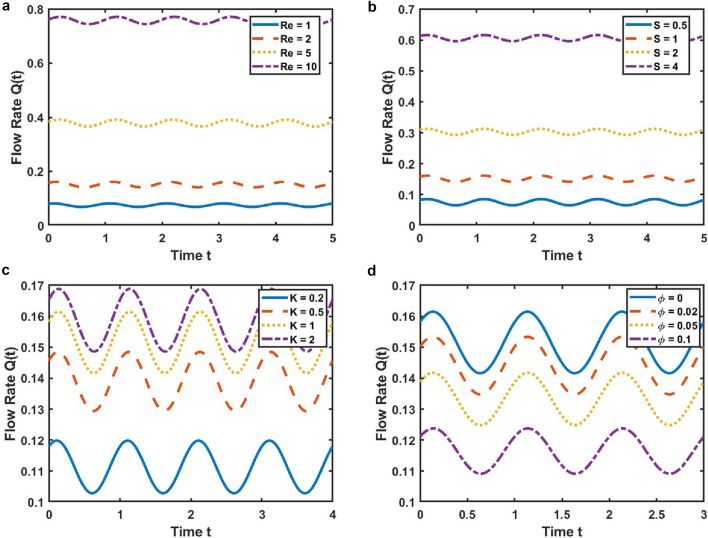
**(a)** Variation of flow rate for different values of Re . **(b)** Variation of flow rate for different values of S . **(c)** Variation of flow rate for different values of K . **(d)** Variation of flow rate for different values of ϕ .

The effect of Reynolds number Re on flow rate is shown in Figure 5a. It is observed that the flow rate increases with increasing Reynolds number. Higher values of Re indicate stronger inertial effects relative to viscous forces, which enhances fluid motion. As a result, the mean flow rate increases significantly with Re, while the oscillatory behavior remains periodic with similar frequency. This confirms that Reynolds number plays a key role in controlling the magnitude of flow.

Figure 5b illustrates the effect of magnetic interaction parameter S on the flow rate. It is clearly observed that the flow rate increases significantly with increasing S. The magnetic field generates a Kelvin force that acts as an additional driving force, accelerating the fluid. As S increases, the entire flow profile shifts upward, while the oscillatory amplitude remains nearly constant. This demonstrates that magnetic forces primarily influence the mean flow rather than the oscillatory characteristics.

The influence of permeability parameter K on flow rate is presented in Figure 5c. It is observed that the flow rate increases with increasing K. Higher permeability reduces the resistance offered by the porous medium, allowing the fluid to flow more freely. For lower values of K, the flow is strongly restricted, resulting in lower flow rates. As K increases, this resistance diminishes, leading to a noticeable increase in the mean flow rate. The oscillatory nature remains unchanged.

The variation of flow rate with time for different values of nanoparticle volume fraction ϕ is shown in Figure 5d. It is observed that the flow rate decreases as ϕ increases. This reduction is due to the increase in effective viscosity of the nanofluid with higher nanoparticle concentration, which enhances resistance to flow. Although the oscillatory nature of the flow remains unchanged, the mean flow rate shifts downward for higher values of ϕ . This indicates that nanoparticle addition slows down the fluid motion while preserving the periodic behavior.

From the above results, it is evident that the flow rate is strongly influenced by nanoparticle concentration, magnetic forces, porous medium properties, and inertial effects. The magnetic interaction parameter and permeability enhance the flow, while increasing nanoparticle concentration reduces it due to increased viscosity.

Importantly, all parameters primarily affect the mean flow level, while the oscillatory nature remains preserved, indicating that the time-dependent forcing governs the periodic behavior, whereas physical parameters control the magnitude of flow.

### 5.5 Wall shear stress

The wall shear stress (Equation 22) is significantly affected by both magnetic and oscillatory effects. The steady shear stress increases with the magnetic parameter due to enhanced flow acceleration near the walls.

**FIGURE 6:**
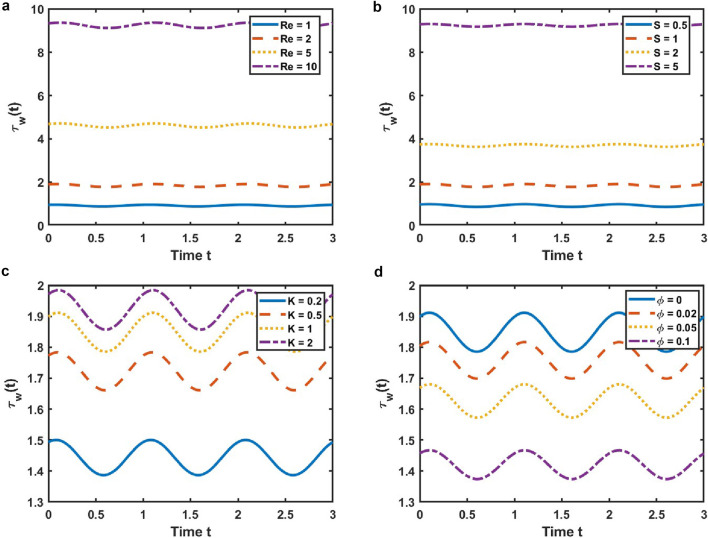
**(a)** Variation of Shear stress for different values of Re . **(b)** Variation of Shear stress for different values of S . **(c)** Variation of Shear stress for different values of K . **(d)** Variation of Shear stress for different values of ϕ .

The variation of wall shear stress with Reynolds number Re is shown in Figure 6a. It is observed that $\tau_{w}$ (t) increases significantly with increasing Re. Higher Reynolds number indicates stronger inertial effects, which enhance fluid motion and increase the velocity gradient at the wall. As a result, the magnitude of wall shear stress increases with Re, while the oscillatory behavior remains periodic.

Figure 6b illustrates the influence of the magnetic interaction parameter S on wall shear stress. It is clearly observed that $\tau_{w}$ (t) increases significantly with increasing S. The applied magnetic field generates a Kelvin force that enhances fluid motion, thereby increasing the velocity gradient at the wall. As S increases, the entire shear stress profile shifts upward, while the oscillatory nature remains unchanged. This confirms that magnetic forces strongly enhance wall shear stress.

The effect of permeability parameter K on wall shear stress is presented in Figure 6c. It is observed that $\tau_{w}$ (t) increases with increasing K. Higher permeability reduces the resistance of the porous medium, allowing greater fluid motion, which leads to higher velocity gradients near the wall. For smaller values of K, the porous medium restricts flow, resulting in lower shear stress. As K increases, this resistance decreases, leading to an increase in wall shear stress.

The variation of wall shear stress $\tau_{w}$ (t) for different values of nanoparticle volume fraction ϕ is shown in Figure 6d. It is observed that the wall shear stress decreases with increasing ϕ . This is because the addition of nanoparticles increases the effective viscosity of the nanofluid, which dampens the velocity gradients near the wall. Although the flow remains oscillatory, the magnitude of shear stress reduces as ϕ increases. This indicates that higher nanoparticle concentration suppresses wall friction effects due to reduced fluid motion.

From the above results, it is clear that wall shear stress is strongly influenced by fluid properties, magnetic forces, porous medium characteristics, and inertial effects. The magnetic interaction parameter, permeability, and Reynolds number enhance the wall shear stress, while increasing nanoparticle concentration reduces it due to increased viscosity. In all cases, the oscillatory nature of shear stress is preserved, indicating that time-dependent forcing governs the periodic behavior, whereas the governing parameters primarily affect the magnitude of shear stress.

## 6 Conclusion

This study investigated the flow and heat transfer characteristics of cerebrospinal fluid (CSF) in a porous channel containing ${\text{Fe}}_{3}{\text{O}}_{4}$ @Au nanofluid under the influence of magnetic forces and magnetic heating. Using appropriate thermophysical properties and a perturbation method, analytical solutions were obtained for velocity, temperature, volumetric flow rate, wall shear stress, and Nusselt number. These results provide insight into the key physical mechanisms governing magnetically influenced CSF transport.

- Velocity and volumetric flow rate increase with permeability, Reynolds number, and magnetic interaction parameter due to reduced resistance and enhanced magnetic driving force, while increasing nanoparticle volume fraction decreases flow because of higher effective viscosity.
- Wall shear stress follows similar trends to velocity, increasing with permeability, Reynolds number, and magnetic parameter, but decreasing with higher nanoparticle concentration.
- Temperature is strongly influenced by magnetic heating, with higher values of the heating parameter $Q^{*}$ leading to significant temperature rise, while higher thermal conductivity reduces temperature by enhancing heat diffusion.
- The effect of nanoparticle volume fraction and Peclet number on temperature is relatively weak compared to magnetic heating and thermal conductivity effects.
- The Nusselt number decreases with increasing magnetic heating parameter due to reduced temperature gradients at the wall, while it increases with thermal conductivity, indicating improved heat transfer capability.
- Peclet number mainly affects the oscillatory behavior of heat transfer, whereas nanoparticle concentration has only a minor influence on the overall heat transfer rate.

The model provides useful insights for optimizing magnetic nanoparticle-based drug delivery and hyperthermia-assisted therapies in the central nervous system. In future work, the model can be extended to include non-uniform nanoparticle distribution, aggregation effects, nonlinear magnetic response, and bioheat transfer mechanisms such as perfusion. Additionally, more realistic geometries of the subarachnoid space and experimental validation can further improve the applicability of the model for clinical and biomedical applications.

## Data Availability

The original contributions presented in the study are included in the article/Supplementary Material, further inquiries can be directed to the corresponding author.
