# Supplementary figures and images for: Mathematical modeling and analysis of magnetic nanoparticle- induced heating in cerebrospinal fluid flow using a core–shell Fe3O4@Au nanoparticles for targeted drug therapy
Source: Front Bioeng Biotechnol. 2026 Jun 25;14:1827203. doi: 10.3389/fbioe.2026.1827203 (PMC13346043; doi:10.3389/fbioe.2026.1827203)

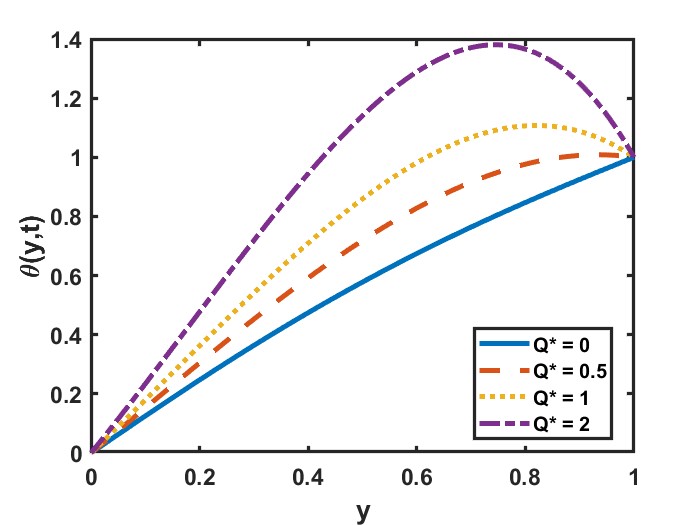

Supplement: Supplementary file 1 [file Image3.jpeg]

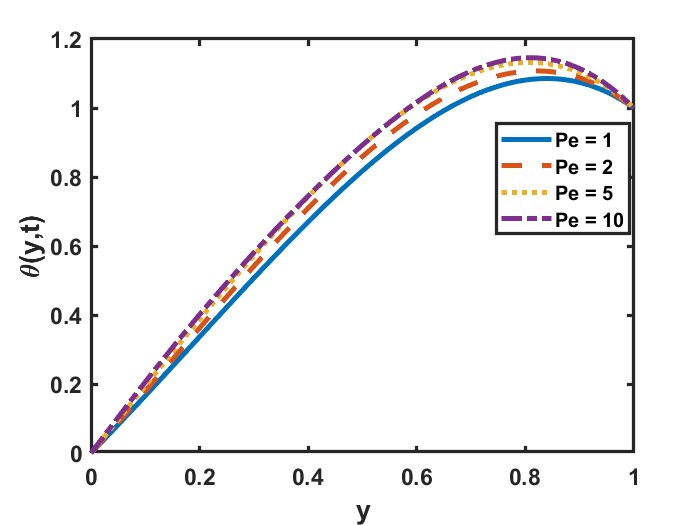

Supplement: Supplementary file 2 [file Image9.jpeg]

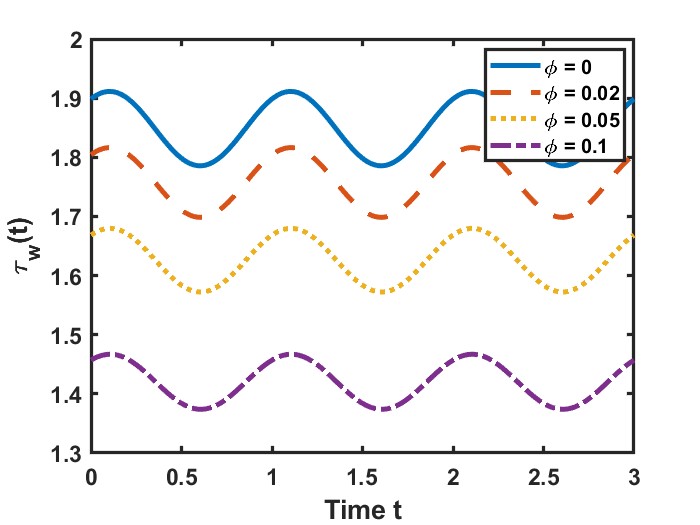

Supplement: Supplementary file 3 [file Image1.jpeg]

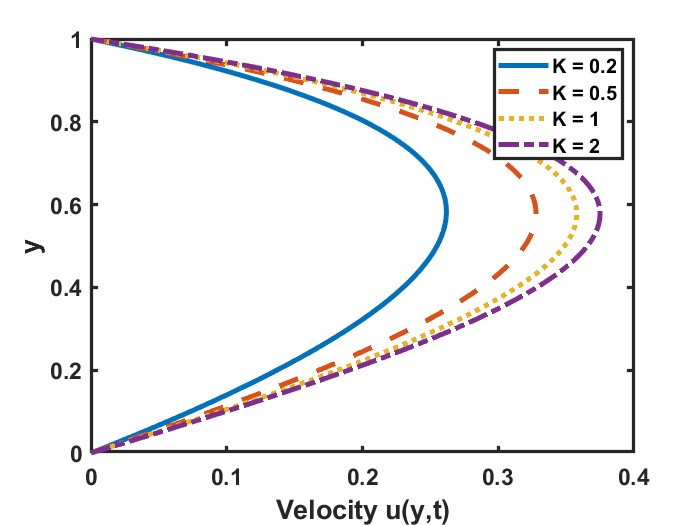

Supplement: Supplementary file 4 [file Image4.jpeg]

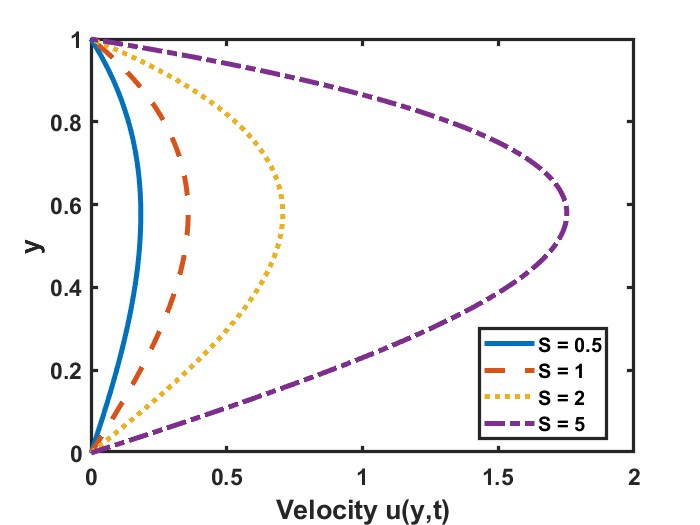

Supplement: Supplementary file 5 [file Image7.jpeg]

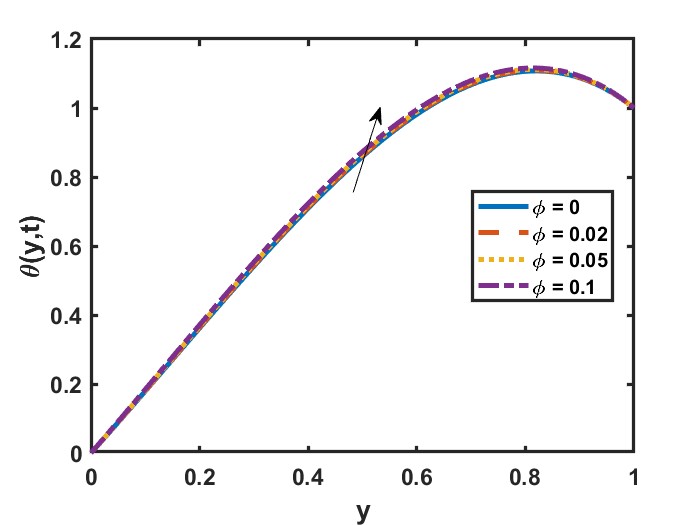

Supplement: Supplementary file 6 [file Image2.jpeg]

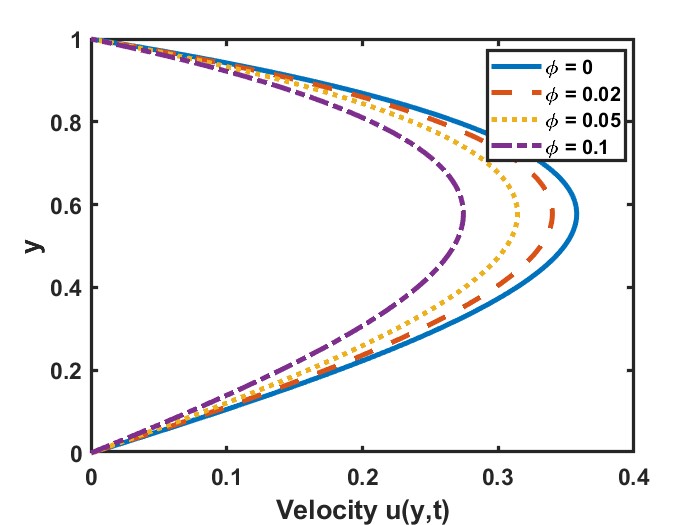

Supplement: Supplementary file 7 [file Image5.jpeg]

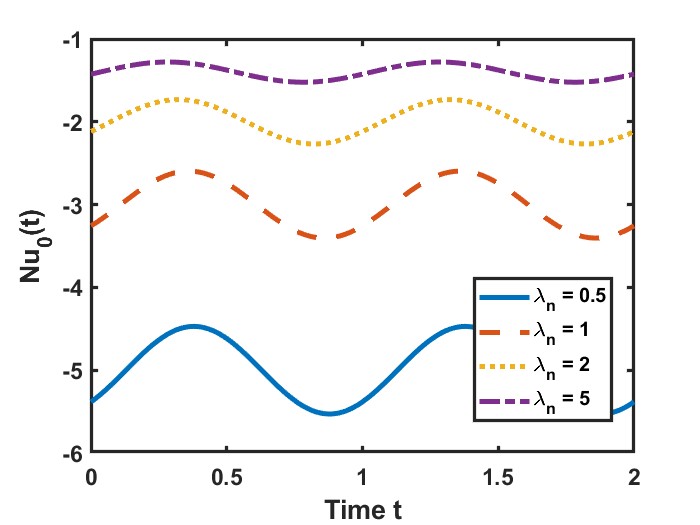

Supplement: Supplementary file 8 [file Image8.jpeg]

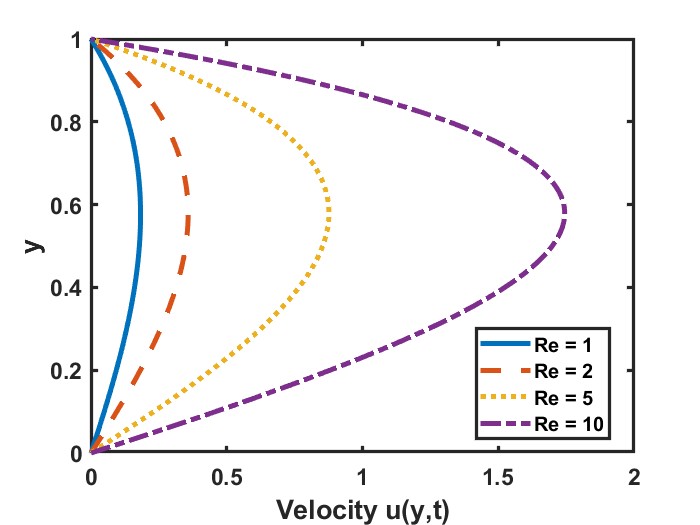

Supplement: Supplementary file 9 [file Image6.jpeg]
